# The Role of the Supine Empty Stress Test in the Evaluation of Women with Stress Urinary Incontinence: A Retrospective Cohort Study

**DOI:** 10.3390/jcm12247697

**Published:** 2023-12-15

**Authors:** Bulut Varlı, Şerife Esra Çetinkaya, Mehmet Murat Seval, Fulya Dökmeci

**Affiliations:** Department of Obstetrics and Gynecology, Ankara University School of Medicine, Ankara 06620, Türkiye; bvarli@ankara.edu.tr (B.V.); seval@ankara.edu.tr (M.M.S.); fdokmeci@gmail.com (F.D.)

**Keywords:** supine empty stress test, stress urinary incontinence, ambulatory urodynamics, abdominal leak point pressure

## Abstract

The International Continence Society recommends the supine empty stress test (SEST) as an accessory test in the evaluation of women with urinary incontinence, especially for the presence of intrinsic sphincter deficiency (ISD). The aim of this study was to investigate the relationship between the SEST and clinical findings in women diagnosed with stress urinary incontinence with single voiding cycle ambulatory urodynamics (AUM). AUM tracings of patients with lower urinary tract symptoms (LUTS = Lower urinary tract symptoms) (*n* = 513) were retrospectively reviewed, and 364 charts with urodynamic SUI were analyzed. Demographics, examination findings, scores of the Sandvik Incontinence Severity Index and validated questionnaires, and AUM findings were compared between SEST-positive and -negative groups. Additionally, the diagnostic accuracy of the SEST in the diagnosis of low abdominal leak point pressure (ALPP ≤ 60 cm H_2_O) in women with pure urodynamic SUI was calculated. The SEST was positive in 41.8% (*n* = 152) of the cohort. Women with a positive SEST had higher scores on the Sandvik severity index (9.2 ± 3.6 vs. 7.5 ± 3.8, *p* = 0.003) and lower ALPP (79.6 ± 29.3 vs. 98.4 ± 31.3, *p* < 0.001). The negative predictive value of the SEST for ISD was found to be 92.4%. Thus, the SEST seems to be an objective clinical test reflecting urinary incontinence severity while excluding the presence of ISD.

## 1. Introduction

Urinary incontinence is defined as the involuntary leakage of urine, with a prevalence of 41% in women older than 40 years of age and has a significant impact on quality of life [1,2]. Stress, urgency, and mixed urinary incontinence are the most common forms of urinary incontinence. Stress urinary incontinence (SUI) is the complaint of involuntary loss of urine upon effort or physical exertion or upon sneezing or coughing [3], which is due to urethral hypermobility and/or intrinsic sphincter deficiency (ISD).

The definition of ISD established by McGuire et al. includes an abdominal leak point pressure (ALPP) ≤ 60 cm H_2_O and/or a maximal urethral closure pressure (MUCP) ≤ 20 cm H_2_O [4]. In 2019, the International Continence Society defined ISD as a very weak urethral closure mechanism [5]. However, in recent years, it has been understood that not only the internal urethral sphincter is responsible for the continence mechanism, but also the entire urethra is involved in the continence mechanism and incontinence develops in case of urethral failure [6]. Large case–control studies that assessed urethral support and function have demonstrated that urethral failure is the primary cause of SUI [7], and epidemiological research has indicated that it is also associated with urgency urinary incontinence (UUI) [8]. The suggested paradigm change emphasizes the necessity of comprehending urethral function and failure, an area of study that has received less attention compared to the assessment of support and detrusor function. Hence, establishing the underlying etiopathogenesis is crucial for successful management planning, together with an optimal counseling approach to fulfill the women’s expectations from the treatment, as women with ISD showed higher treatment failure rates after anti-incontinence surgery with midurethral slings [9].

Among the recommended evaluation steps for SUI in women, the cough stress test (CST) is mandatory for the demonstration of SUI before any anti-incontinence surgery; the visualization of fluid loss from the urethra simultaneously with a cough is considered diagnostic for SUI [10,11]. The ICS described the ICS-uniform CST in 2018 because the standards for CST were not clear and additionally mentioned the supine empty stress test (SEST) as an accessory test to evaluate the existence of ISD [12]. This suggestion came from the fact that a positive SEST was linked to lower ALPP and MUCP in earlier studies [13,14]; however, not all have found the same relationship between the urodynamic measures of urethral function and clinical measures, including the SEST [15,16].

The abdominal leak point pressure is defined as the lowest value of intentionally raised intravesical pressure that provokes urinary leakage in the absence of detrusor contraction. It has been reported to be suggestive of poor urethral function [4] and has been used in urodynamic studies to discriminate between ISD and urethral hypermobility in women with SUI. It has also been reported to be important in the prediction of recurrent urinary incontinence after midurethral sling surgery [17,18]. In a 12-year follow-up study after a tension-free vaginal tape procedure, a VLPP < 60 cm H_2_O was the only independent predictor of recurrence. However, the biggest obstacle in the determination of ALPP is the need for an invasive urodynamic study (UDS), which appears to be used less frequently to evaluate SUI due to cost, personal shortage, or other reasons [19]. According to previous research, the SEST has been suggested as a noninvasive and low-cost alternative to UDS in the prediction of ALPP; however, the results are conflicting.

In this study, we aimed to investigate the relationship between the SEST and both subjective and objective clinical findings in women with SUI at a single voiding cycle ambulatory urodynamic evaluation and to reveal the clinical predictive value of the SEST for poor urethral function in terms of low ALPP.

## 2. Materials and Methods

### 2.1. Study Design

In this retrospective cohort study, the records of patients with lower urinary tract symptoms (LUTSs) who underwent AUM in the urogynecology unit of the Ankara University Faculty of Medicine, Department of Obstetrics and Gynecology, between January 2015 and December 2022, were reviewed. The inclusion criteria for the study were age ≥ 18 years, presence of SUI at AUM, and negative urine culture. The exclusion criteria were AUM tracings with incomplete filling (cystometric capacity < 150 mL) and/or inadequate pressure–flow phases, high postvoid residual urine volume (PVR > 100 mL), and leakage only with detrusor overactivity (DO). Urodynamic recordings with either pure SUI or SUI and concomitant urinary leakage with DO were included in the final analysis. Data were grouped according to the presence of the SEST at the records of clinical examination and were compared between the SEST-positive and -negative groups. Additionally, the accuracy of SEST in diagnosing intrinsic sphincter deficiency (in terms of ALPP < 60 cm H_2_O) in women with pure urodynamic SUI was investigated; the sensitivity, specificity, and negative and positive predictive values of the SEST for an ALPP < 60 cm H_2_O were calculated. The Institutional Review Board (Decision No. 20, Dated 23 August 2023) approved the study’s design, and because it was a retrospective study, the patients’ written consent was not required.

### 2.2. Study Parameters

Data regarding demographics, patient-reported LUTS, clinical findings, Sandvik incontinence severity index [20], Turkish-validated short forms of the Urinary Distress Inventory (UDI-6), Incontinence Impact Questionnaire (IIQ-7) [21], Overactive Bladder Awareness Tool-8 (OAB-V8) [22], Pelvic Organ Prolapse/Urinary Incontinence Sexual Questionnaire-12 (PISQ-12) [23], and single voiding cycle AUM findings were retrieved. Clinical findings included the 3-day voiding diary, Q-tip test, CST, SEST, simplified POP-Q staging, and assessment of pelvic floor muscle strength. As part of the routine urogynecologic examination in our unit, the CST was performed with a comfortably full bladder before the evaluation with uroflowmetry. By adding the voided volume at uroflowmetry and the PVR volume measured with a catheter, it was possible to calculate the cystometric capacity at CST. The SEST was performed in the lithotomy position 5 min after voiding. The assessments of pelvic floor muscle strength (Modified Oxford Scale) and simplified POPQ staging were performed as previously described [3,24]. We used the LUNA ambulatory monitoring recorder (MMS^TM^, Enschede, Netherlands) for ambulatory urodynamic monitoring during a single voiding cycle as the main method for urodynamic examination in our urogynecology unit. It was performed according to a standard protocol set out in a previous study [25] and the criteria set by the ICS subcommittee for AUM [26]. In patients with advanced-stage pelvic organ prolapse (stages III–IV), AUM was performed with vaginal packing for the reduction of prolapse. The leak point pressure (LPP) is defined as the pressure value monitored on the AUM trace simultaneously at which the urinary incontinence button was pressed by the patient on the LUNA event tracker while performing maneuvers to increase abdominal pressure. In the tracings of AUM, the lowest change in the intravesical pressure associated with patient-reported leakage was used as the ALPP value [3].

### 2.3. Statistical Analysis

Statistical analyses were performed using SPSS Statistics for Windows, Version 20.0. Armonk, NY, USA: IBM Corp. [27]. The variables were analyzed using both analytical techniques, such as the Kolmogorov–Smirnov and Shapiro–Wilk tests, as well as visual techniques, such as histograms and probability plots, to assess the distribution of data for a normal distribution. The mean ± standard deviation (SD) and median (range) were used to display continuous variables, whereas categorical variables were presented as numbers and percentages. In the comparison of continuous variables between the SEST-positive and -negative groups, a parametric test (Student’s *t*-test) was used to compare variables with a normal distribution and a nonparametric test (Mann–Whitney U-test) was used to compare variables, which do not have a normal distribution. The chi-square test or Fisher’s exact test was used to compare categorical variables between the groups, and Fisher’s exact test was preferred if there was a sample size less than five on a cell. Diagnostic accuracy of SEST for an ALPP < 60 cm H_2_O was calculated with 2 × 2 table of true-positive, true-negative, false-positive, and false-negative values. The subsequent criteria were established for determination of these values:

- A true-positive finding was a positive SEST with an ALPP ≤ 60 cm H_2_O.
- A false-positive finding was a positive SEST with an ALPP > 60 cm H_2_O.
- A false-negative finding was a negative SEST with an ALPP ≤ 60 cm H_2_O.
- A true-negative finding was a negative SEST with an ALPP > 60 cm H_2_O.

## 3. Results

**Figure 1:**
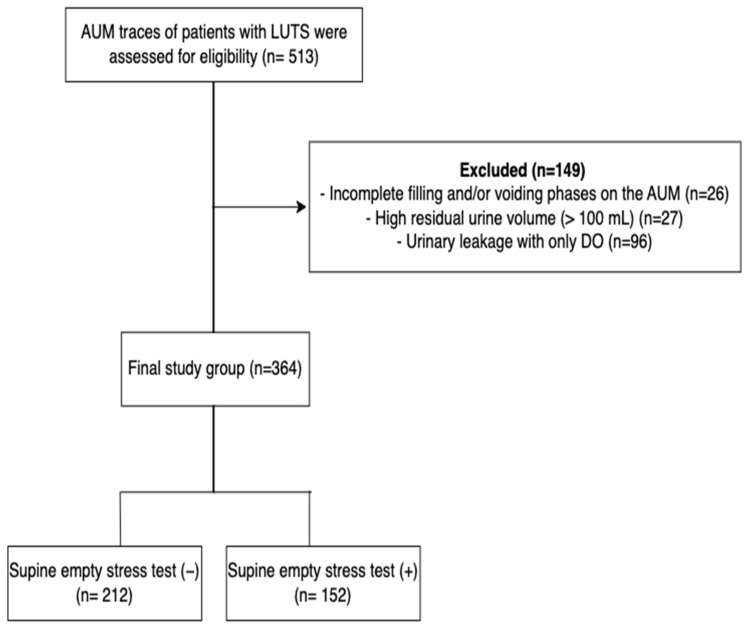
Flow chart of the selection of the study population (AUM: Ambulatory urodynamic monitoring; DO: Detrusor overactivity; LUTSs: Lower urinary tract symptoms).

During the study period, 513 AUMs were performed in women with LUTS. Charts with incomplete filling and/or voiding phases (*n* = 26), high residual urine volume (PVR > 100 mL) (*n* = 27), and urinary incontinence associated only with DO (*n* = 96) were excluded. Data from 364 women with either pure urodynamic SUI (39.8%, *n* = 145) or SUI + urinary incontinence with DO (60.1%, *n* = 219) were evaluated in the final analysis (Figure 1).

The mean age was 53.3 ± 10.6 (23–88) years; the mean body mass index (BMI) was 30.3 ± 5.1 (18–48) kg/m2; 53% (*n* = 193) were postmenopausal; and 14.6% (*n* = 53) had POPQ stage ≥ 3 prolapse in any compartment. A history of anti-incontinence surgery was present in 6.3% (*n* = 23) of the women, and the transobturator tape procedure was the most common (69.6%, *n* = 16).

The SEST was positive in 41.8% (*n* = 152) and negative in 58.2% (*n* = 212) of women. The groups were comparable in terms of age, parity, BMI, smoking status, or previous surgery for incontinence (*p* > 0.447). However, significantly more women in the SEST-negative group were postmenopausal (57.5% vs. 46.7%, *p* = 0.036). In the SEST-positive group, significantly more women reported SUI (100% vs. 85%, *p* < 0.001), postural (46.2% vs. 25%, *p* < 0.001), and coital incontinence (31.5% vs. 14%, *p* < 0.001) on direct questioning and were found to have significantly more daily urinary incontinence episodes (*p* < 0.001) according to the 3-day voiding diary. At physical examination, the positive Q-tip test and CST rates were significantly higher in the SEST-positive group (87.5% vs. 76.9%, *p* = 0.015 and 100% vs. 58%, *p* < 0.001, respectively), whereas significantly more women in the SEST-negative group had POPQ stage ≥ 3 anterior (14.2% vs. 5.2%, *p* = 0.01) and apical (9.4% vs. 3.9%, *p* = 0.046) prolapse (Table 1).

According to the UDI-6, women with a positive SEST had significantly higher stress subscale scores (87.5 (0–100) vs. 75 (0–100), *p* < 0.001). They also had higher scores on the Sandvik severity index (9.2 ± 3.6 vs. 7.5 ± 3.8, *p* = 0.003), and more women had a Sandvik severity index score of eight or more (74.3% vs. 54.2%, *p* < 0.001). However, there was no significant difference between the groups in terms of quality of life or sexual function (Table 2).

**Figure 2:**
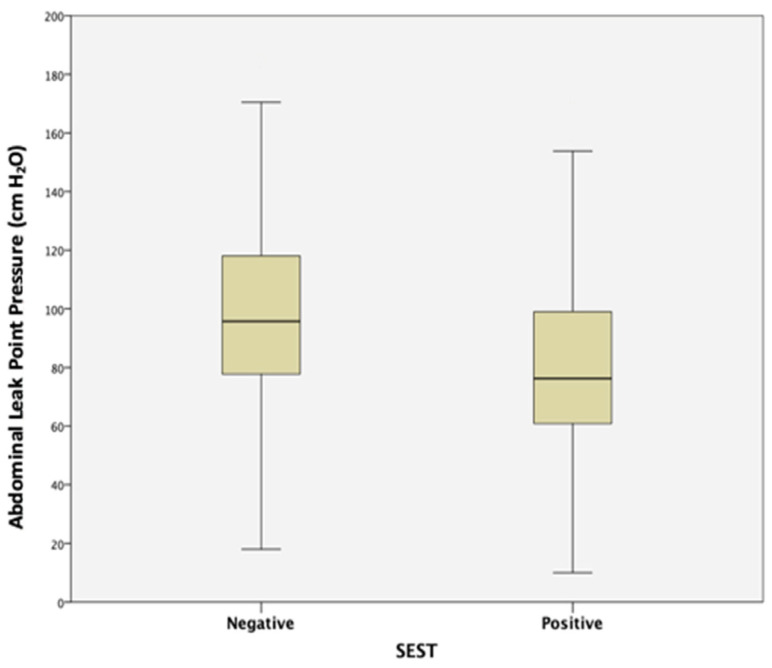
Comparison of abdominal leak point pressure values in women with negative and positive SESTs.

On comparison of the cystometry findings between the groups, maximum cystometric capacity was significantly higher (477.8 ± 207.2 vs. 414.7 ± 198.1, *p* = 0.003) and ALPP was significantly lower (79.6 ± 29.3 vs. 98.4 ± 31.3, *p* < 0.001) in women with a positive SEST (Figure 2). The number of urinary incontinence episodes (11.2 ± 8.5 vs. 8.7 ± 9, *p* < 0.001) and increase in pad weight (49.4 ± 55.4 vs. 40.4 ± 57.9, *p* = 0.005) were also significantly higher in SEST-positive women. In the pressure–flow study, the voided volume was significantly higher (416.4 ± 201.9, *p* = 0.003), and the detrusor pressure at maximum flow rate (PdetQmax) was significantly lower (31.9 ± 17.6 vs. 40.9 ± 37.5, *p* = 0.026) in the same group (Table 3).

The diagnostic accuracy of the SEST for a low ALPP was evaluated in 145 women with pure urodynamic SUI at AUM. The sensitivity and specificity were 64.7% and 57%, respectively. Whereas 17 women had an ALPP < 60 cm H_2_O, the positive predictive value was 16.6%, and the negative predictive value of a negative SEST for an ALPP < 60 cm H_2_O was 92.4% (Table 4).

## 4. Discussion

In this study, a positive SEST was found to be associated with patient-reported coital and postural urinary incontinence symptoms, higher bother of patients from SUI symptoms in the SEST (+) group, and more severe urinary incontinence. Objective clinical measures were found to be in accordance; these women had more urinary incontinence episodes and higher increases in pad weight in the voiding diary at urodynamic monitoring.

Our results are in line with studies reporting the SEST as a clinical measure of SUI severity [15,28]. Nager et al. also evaluated the relationship between the SEST and measures of urinary incontinence severity and urethral function among the participants of the Trial of Mid-Urethral Slings (TOMUS) study, which included 597 women with pure or stress-predominant urinary incontinence [15,29]. They reported that the objective measures of severity, 24 h pad weight, and daily urinary incontinence episodes were significantly higher and clinically meaningful in the SEST-positive group; however, although the Medical Epidemiological and Social Aspects of Aging (MESA) total and stress scores were significantly higher in the same group of women, they suggested that the subjective measures of severity were not clinically relevant because the difference in the scores was low [15]. We additionally used the Sandvik incontinence severity index as a subjective measure, which was described by Sandvik et al. as a scoring system validated by the 48 h pad weight test, to be more objective [20]. In the present study, SEST-positive women’s scores were significantly higher on the Sandvik severity index, and 74.3% of these women had a score ≥ 8 points, indicating more severe incontinence. However, we were not able to show the reflection of severity on incontinence-related quality of life and sexual function, contrary to the results of Nager et al., who reported a significant difference in IIQ scores in women with a positive SEST [15].

In this study, a positive SEST result was associated with more urinary incontinence episodes, higher pad weight, higher maximum cystometric capacity, lower ALPP and PdetQmax, and similar frequencies of DO at AUM. Although urodynamic parameters were significantly different between the groups, the UDI-6 and IIQ-7 scores were comparable between the groups, which suggests that the implications of these parameters on quality of life appear clinically insignificant.

Previous studies investigating the relationship between the SEST and various urodynamic measures, including urethral function, have reported conflicting results [13,14,15,30]. Nager et al. reported no association between the SEST and either MUCP or VLPP in their study with TOMUS trial participants [15]. On the other hand, there are studies supporting an association; Lobel and Sand performed one of the earlier studies evaluating the role of the SEST in the prediction of lSD, evaluating 304 women with urinary incontinence who underwent urodynamics, of whom 78% were found to have genuine SUI, and reported that women with a positive SEST had significantly lower MUCP values with similar rates of DO and similar maximum cystometric capacity [13]. In their study of 179 women with either genuine SUI alone or with DO at urodynamics, McLennan and Bent found a significant relationship between a low VLPP (<60 cm H_2_O) and a positive SEST [14]. In the sub-group analysis of the Stress Incontinence Surgical Treatment Efficacy Trial (SISTEr) [16,31], Albo et al. also discovered that VLPP values were significantly lower in a group of patients with a positive SEST (mean 75 vs. 85 cm H_2_O, *p* = 0.011). They evaluated 655 women and said that this significant difference was not clinically meaningful [16]. In our study, ALPP was similarly statistically lower in women whose SEST was positive, with quite similar median values between the SEST-negative and SEST-positive groups (76.2 vs. 95.7 cm H_2_O, *p* < 0.001), but had wide overlapping ranges, making clinical interpretation and discrimination difficult (18–186 vs. 10–171 cm H_2_O). In contrast, Arribillaga et al. performed one of the latest studies showing a relationship between ALPP and the SEST and developed a clinical scoring system to predict ALPP < 60 cm H_2_O for women with SUI. In their study, the SEST was found to be positive in 75.8% (22/29) of women with low ALPP, and on multivariate analysis, a positive SEST was found to be an independent predictor of an ALPP < 60 cm H_2_O [OR:3.14 CI 95% (1.11–8.85, *p* = 0.03)] [30].

In the present cohort, SEST-positive women had significantly lower PdetQmax and ALPP values, suggesting lower urethral resistance. It is noteworthy to emphasize that women with a negative SEST had a significantly higher rate of anterior vaginal wall (*p* = 0.01) and apical compartment prolapse (*p* = 0.046). However, prolapse reduction was routinely performed during AUM. Chai et al. explored the correlation between clinical and urodynamic parameters in patients in the TOMUS trial [32]. PdetQmax was the only urodynamic parameter that was significantly and positively linked to urethral function in terms of both MUCP and VLPP. This shows that higher voiding pressures are needed to overcome higher urethral resistance. Age, BMI, history of incontinence surgery, and Q-tip delta were clinical parameters found to be correlated with measures of urethral function in their study [33]. Although nearly half of the study population was obese (mean BMI: 30.3 ± 5.1 kg/m^2^), age, BMI, and history of incontinence surgery were found to be similar among the groups in this cohort, whereas the rate of women with urethral hypermobility was higher in SEST-positive women.

Even though the SEST seems to be linked to urethral function, its diagnostic accuracy and predictive value for ISD are still limited. This is because its sensitivity and positive predictive values have been found to be low in this and other studies. In our study, the SEST had a negative predictive value of 92.4% for an ALPP < 60 cm H_2_O. This is similar to other studies in the literature that found negative predictive values between 76 and 95% [13,14,33,34], which supports its use as a clinical tool to rule out ISD, as suggested [35].

The current study has several limitations. First, it has the inherent bias of a retrospective design including patient selection bias. In addition, clinical examinations were performed without pelvic organ prolapse reduction, while AUM was performed with prolapse reduction. In future prospective studies, designing the examination of women both with and without prolapse reduction may clarify the relationship between advanced-stage prolapse and the SEST. The mean maximum cystometric capacity was higher in women who were positive for the SEST. This could have influenced the results, but the mean and median cystometric capacity for these women was 480 mL, which is an acceptable value. Lowenstein et al. reported that urine volume on the bladder during leakage can range from 100 to 400 mL or greater, with 35% of women leaking at a volume of ≥400 mL, and urine volume on the bladder did not affect the median VLPP and MUCP values [36]. Another limitation may be that we did not know the bladder volume during leakage as we studied the AUM data; however, the use of AUM may also be expressed as a strength, as it is a more sensitive tool with the advantage of physiological bladder filling and the evaluation of leakage during daily activities. Moreover, we investigated the relationship between the SEST and clinical measures of incontinence severity and urodynamics in all aspects. To the best of our knowledge, this is the first study evaluating the diagnostic accuracy of the SEST in women who underwent ambulatory urodynamic monitoring.

## 5. Conclusions

In conclusion, the SEST was found to be an objective clinical test reflecting urinary incontinence severity while excluding the presence of ISD, thus providing valuable in-formation for the initial evaluation of women with urinary incontinence. However, the low sensitivity and positive predictive value of the test limit its diagnostic performance regarding urethral function, and multichannel urodynamics are still needed to better understand the pathophysiology of SEST-positive women. Treatment outcomes based on the SEST results have not been reported in the literature, and the predictive value of the SEST on treatment outcomes needs further investigation.

## Figures and Tables

**Table 1:** Comparison of demographics, patient-reported LUTS, and clinical findings of women with negative and positive SESTs.

Demographics	SEST (−) (*n* = 212)	SEST (+) (*n* = 152)	*p*
Age (years)			0.381
Mean (SD)	53.3 (11.2)	52.4 (9.1)	
Median (min–max)	53 (30–88)	51 (26–78)	
BMI (kg/m^2^)			0.447
Mean (SD)	30.1 (4.9)	30.5 (5.2)	
Median (min–max)	30 (18–45)	30 (21–48)	
Postmenopausal, *n* (%)	122 (57.5)	71 (46.7)	0.036
Parity (*n*)			0.729
Mean (SD)	2.9 (1.9)	2.8 (1.8)	
Median (min–max)	2 (0–14)	2 (0–11)	
Smoker, *n* (%)	40 (18.9)	29 (19.1)	0.953
Previous anti-incontinence surgery, *n* (%)	12 (5.7)	11 (7.2)	0.654
Patient-reported LUTS, *n* (%)			
Frequency	159 (75)	115 (75.7)	0.886
Urgency	164 (77.4)	112 (73.7)	0.419
Nocturia	157 (74.1)	108 (71.1)	0.436
Suprapubic pain	104 (49.1)	68 (44.7)	0.410
SUI	180 (85)	152 (100)	<0.001
UUI	163 (76.9)	106 (69.7)	0.306
Postural UI	53 (25)	70 (46.2)	<0.001
Coital UI	28/200 (14)	47/149 (31.5)	<0.001
3-day bladder diary findings, median (min–max)			
Daily fluid intake, (L)	2.1 (0.5–5.7)	2.1 (0.6–5.6)	0.652
Daily micturition episodes, (*n*)	8 (1–35)	8 (2–18)	0.757
Daily UI episodes, (*n*)	3 (0–22)	3 (0–20)	0.003
Physical examination findings			
POPQ ≥ Stage III			
Anterior, *n* (%)	30 (14.2)	8 (5.2)	0.010
Apical, *n* (%)	20 (9.4)	6 (3.9)	0.046
Posterior, *n* (%)	15 (7.1)	7 (4.6)	0.292
Positive Q-tip test, *n* (%)	163 (76.9)	133 (87.5)	0.015
Positive CST, *n* (%)	123 (58)	152 (100)	<0.001
PFMS (MOS), mean (SD)	2.2 ± 0.9	2.0 ± 0.9	0.39

BMI: Body mass index; LUTS: Lower urinary tract symptoms; MOS: Modified Oxford Grading scale; PFMS: Pelvic floor muscle strength; SEST: Supine empty stress test; SUI: Stress urinary incontinence; UI: Urinary incontinence; UUI: Urgency urinary incontinence. *p* < 0.05 considered statistically significant.

**Table 2:** Comparison of the scores of the questionnaires in women with negative and positive SESTs.

Scores of Questionnaires	SEST (−) (*n* = 212)	SEST (+) (*n* = 152)	*p*
Sandvik incontinence severity index,			<0.001
Mean (SD)	7.5 (3.8)	9.2 (3.6)	
Median (min–max)	8 (1–12)	12 (1–12)	
Sandvik incontinence severity index ≥ 8,	115 (54.2)	113 (74.3)	<0.001
*n* (%)			
UDI-6			
Total,			0.723
Mean (SD)	58.3 (22.8)	59.1 (27.9)	
Median (min–max)	62.5 (8–100)	62.5 (4–100)	
Irritative subscale,			0.243
Mean (SD)	71.9 (27.9)	68 (33)	
Median (min–max)	75 (0–100)	75 (0–100)	
Stress subscale,			<0.001
Mean (SD)	65 (29.5)	75.9 (29.3)	
Median (min–max)	75 (0–100)	87.5 (0–100)	
Obstructive subscale,			0.207
Mean (SD)	37.8 (31.4)	33.5 (32.8)	
Median (min–max)	37.5 (0–100)	37.5 (0–100)	
IIQ-7 total,			0.070
Mean (SD)	45.7 (29.7)	51.6 (31.1)	
Median (min–max)	51.8 (0–100)	61.2 (0–100)	
OAB-V8 total,			0.671
Mean (SD)	22.3 (10.8)	22.8 (10.5)	
Median (min–max)	24 (0–40)	23 (0–40)	
PISQ-12 total,			0.876
Mean (SD)	21.3 (13.1)	21.1 (13.5)	
Median (min–max)	25 (9–42)	24.5 (6–46)	

IIQ-7: Short form of the Incontinence Impact Questionnaire; OAB-V8: Overactive bladder-validated 8-question; PISQ-12: Short form of the Pelvic Organ Prolapse/Urinary Incontinence Sexual Questionnaire; SEST: Supine empty stress test; UDI-6: Short form of the Urinary Distress Inventory. *p* < 0.05 considered statistically significant.

**Table 3:** Comparison of AUM findings in women with negative and positive SESTs.

Cystometry	SEST (−) (*n* = 212)	SEST (+) (*n* = 152)	*p*
Duration (minutes)			0.561
Mean (SD)	92.3 (30.3)	93.1 (23.9)	
Median (min–max)	84 (43–255)	85 (52–242)	
Maximum cystometric capacity (mL)			0.003
Mean (SD)	414.7 (198.1)	477.8 (207.2)	
Median (min–max)	377 (155–1114)	479.5 (152–1033)	
Detrusor overactivity, *n* (%)	115 (54.2)	79 (52.2)	0.464
Number of incontinence episodes (*n*)			<0.001
Mean (SD)	8.7 (9)	11.2 (8.5)	
Median (min–max)	5 (1–70)	9 (1–48)	
Increase in pad weight, (grams)			0.005
Mean (SD)	40.4 (57.9)	49.4 (55.4)	
Median (min–max)	14 (0.9–300)	26 (1.2–250)	
Abdominal leak point pressure, (cm H_2_O)			<0.001
Mean (SD)	98.4 (31.3)	79.6 (29.3)	
Median (min–max)	95.7 (18–186)	76.2 (10–171)	
Pressure–flow study			
Voided volume (mL)			0.003
Mean (SD)	355.8 (183.1)	416.4 (201.9)	
Median (min–max)	332 (140–912)	433.5 (141–969)	
PVR (mL)			0.591
Mean (SD)	53.1 (45.9)	53.7 (24.7)	
Median (min–max)	50 (5–90)	50 (10–95)	
Qmax (mL/s)			0.152
Mean (SD)	29.9 (14.1)	33.2 (15.3)	
Median (min–max)	39 (2–72)	32.5 (2–74)	
Pdet Qmax (cm H_2_O)			0.026
Mean (SD)	40.9 (37.5)	31.9 (17.6)	
Median (min–max)	31.5 (8–91)	26.4 (3–94)	
Flow time, (seconds)			0.849
Mean (SD)	38.7 (15.4)	37.4 (16.6)	
Median (min–max)	29.5 (9–74)	26 (8–67)	

PdetQmax: Detrusor pressure at maximum flow; PVR: Postvoid residual urine volume; Qmax: Maximum flow rate; SD: Standard deviation; SEST: Supine empty stress test. *p* < 0.05 considered statistically significant.

**Table 4:** Diagnostic accuracy of SEST in predicting ALPP ≤ 60 cm H_2_O in women with urodynamic pure SUI.

	ALPP ≤ 60 cm H_2_O (*n =* 17)	ALPP > 60 cm H_2_O (*n =* 128)
**SEST (+) (*n* = 66)**	11	55
**SEST (−) (*n* = 79)**	6	73
Sensitivity	64.7%	
Specificity	57%	
Positive predictive value	16.6%	
Negative predictive value	92.4%	

ALPP: Abdominal leak point pressure; SEST: Supine empty stress test.

## Data Availability

Data available upon reasonable request from corresponding author due to privacy and ethical restrictions.
